# Lumbar segmental mobility disorders: comparison of two methods of defining abnormal displacement kinematics in a cohort of patients with non-specific mechanical low back pain

**DOI:** 10.1186/1471-2474-7-45

**Published:** 2006-05-19

**Authors:** J Haxby Abbott, Julie M Fritz, Brendan McCane, Barry Shultz, Peter Herbison, Brett Lyons, Georgia Stefanko, Richard M Walsh

**Affiliations:** 1 Director, Clarity Clinical Research Consultants, Dunedin, New Zealand; 2 Division of Physical Therapy, College of Health, University of Utah, 520 Wakara Way, Salt Lake City, UT 84108, USA; 3 Computer Science Department, University of Otago, PO Box 56, Dunedin, New Zealand; 4 College of Health, University of Utah, Salt Lake City, UT 84108, USA; 5 Department of Preventive and Social Medicine, University of Otago, PO Box 913, Dunedin, New Zealand; 6 Radiology Department, Southland Hospital, Southland District Health Board, Ivercargill, New Zealand; 7 Dunedin Hospital, Otago District Health Board, Dunedin, New Zealand; 8 Dunedin School of Medicine, University of Otago, Dunedin, New Zealand

## Abstract

**Background:**

Lumbar segmental rigidity (LSR) and lumbar segmental instability (LSI) are believed to be associated with low back pain (LBP), and identification of these disorders is believed to be useful for directing intervention choices. Previous studies have focussed on lumbar segmental rotation and translation, but have used widely varying methodologies. Cut-off points for the diagnosis of LSR & LSI are largely arbitrary. Prevalence of these lumbar segmental mobility disorders (LSMDs) in a non-surgical, primary care LBP population has not been established.

**Methods:**

A cohort of 138 consecutive patients with recurrent or chronic low back pain (RCLBP) were recruited in this prospective, pragmatic, multi-centre study. Consenting patients completed pain and disability rating instruments, and were referred for flexion-extension radiographs. Sagittal angular rotation and sagittal translation of each lumbar spinal motion segment was measured from the radiographs, and compared to a reference range derived from a study of 30 asymptomatic volunteers. In order to define reference intervals for normal motion, and define LSR and LSI, we approached the kinematic data using two different models. The first model used a conventional Gaussian definition, with motion beyond two standard deviations (2sd) from the reference mean at each segment considered diagnostic of rotational LSMD and translational LSMD. The second model used a novel normalised within-subjects approach, based on mean normalised contribution-to-total-lumbar-motion. An LSMD was then defined as present in any segment that contributed motion beyond 2sd from the reference mean contribution-to-normalised-total-lumbar-motion. We described reference intervals for normal segmental mobility, prevalence of LSMDs under each model, and the association of LSMDs with pain and disability.

**Results:**

With the exception of the conventional Gaussian definition of rotational LSI, LSMDs were found in statistically significant prevalences in patients with RCLBP. Prevalences at both the segmental and patient level were generally higher using the normalised within-subjects model (2.8 to 16.8% of segments; 23.3 to 35.5% of individuals) compared to the conventional Gaussian model (0 to 15.8%; 4.7 to 19.6%). LSMDs are associated with presence of LBP, however LSMDs do not appear to be strongly associated with higher levels of pain or disability compared to other forms of non-specific LBP.

**Conclusion:**

LSMDs are a valid means of defining sub-groups within non-specific LBP, in a conservative care population of patients with RCLBP. Prevalence was higher using the normalised within-subjects contribution-to-total-lumbar-motion approach.

## Background

Many authors have postulated that abnormal kinematic behaviour of the lumbar spine is associated with low back pain (LBP) [1-4]. Abnormally limited or excessive sagittal dispacement are the lumbar segmental mobility disorders (LSMDs) most commonly referred to in the literature, and are respectively referred to as lumbar segmental rigidity (LSR) and lumbar segmental instability (LSI) [3-6]. Researchers have used various criteria for identifying abnormal kinematics in groups of patients with LBP, with the most common criteria being radiographically measurable abnormalities in the magnitude of sagittal plane rotation and translation. To date, however, there has not been a consensus among authors regarding either the methodology for measuring motion, or the cut-off value or values beyond which the motion segment should be diagnosed as having a LSMD [2,7-22]. Many studies in the literature use differing and non-standard patient positioning for radiography, such as clamping the pelvis during standing flexion-extension, applying overpressure to the trunk, or F-E radiography in the sitting position [7,21,23-25]. There are a multiplicity of methods for defining reference marks on the radiographic images of vertebrae, and for measuring rotation and translation, the properties of which are widely variable [26]. As a result, authors have arbitrarily nominated values based very loosely on a combination of clinical opinion and what scant research data existed at the time of publication. This has been the major shortcoming in the rigour of the LSMD literature since the earliest observational reports.

A second shortcoming in the definitions of both LSI and LSR is that they are typically diagnosed at the segmental level, simply by comparing the motion value recorded at each segment to an arbitrary cut-off value for that segmental level, level by level, without regard to the motion of the neighbouring segments within an individual patient. A more appropriate approach may be to identify a segment or segments within an individual that exhibit significantly different kinematics to neighbouring segments. Hence, in a patient with LBP, one segment may exhibit substantially greater displacement in comparison to other segments within that patient's lumbar spine. Conversely, another patient with LBP may have one segment that contributes very little motion, while three neighbouring segments move through a generous range. How much discrepency between neighbouring segments within an individual should be considered normal? Do such discrepancies in within-subject motion constitute valid LSMDs?

In biomedical science there are six ways of defining normal, with regard to an observation, test or measure [27,28]. These are presented in Table 1. As previous research suggests that the kinematic parameters of spinal segmental motion conform to a normal (Gaussian) distribution, the Gaussian definition is an appropriate one to apply to such data [29]. Because little is known about the association of these kinematic parameters with risk of LBP (definitions 3 & 5, Table 1), or with outcome attributable to therapy (definition 4, Table 1), the Gaussian definition is arguably the most appropriate method of defining normal and abnormal motion. Using a Gaussian definition of abnormality [27], lumbar segmental motion can be considered abnormal if kinematic measurements fall outside an established reference range. The reference range is defined by the two standard deviation (2sd) limits from normal mean values, provided those mean values had been established from a suitably representative sample of asymptomatic individuals [27].

In order for LSMDs to be considered valid clinical entities they must also be associated with the symptom of LBP. To establish the validity of a Gaussian approach to defining abnormal, it is essential to establish that a) the distribution of the variable measured conforms to a normal (Gaussian) distribution, and b) the population with the target disease has significantly different values of the measured variable, in comparison to a population without the disease. A significantly higher prevalence of abnormal lumbar displacement kinematics in a prospective cohort of patients with LBP, than would be expected in a population of asymptomatic individuals, would be evidence in support of LSMDs being valid diagnostic entities [13]. Non-probability sampling of the LBP population of interest, such as retrospective and non-consecutive samples of convenience, are not sound evidence for estimating prevalence or patterns of LSMDs. The few studies of prevalence that have used a consecutive patient cohort design have either not used an appropriate asymptomatic comparison group, or have used differing methods of radiographic measurement, or arbitrary definitions of abnormal motion, some of which have since been questioned or abandoned. To date, there has not been a prospective cohort study of consecutive non-surgical patients to establish prevalence of LSMDs in primary care.

The purpose of this paper is to compare a conventional between-subjects Gaussian approach to characterising LSMDs to a novel Gaussian approach using within-subject normalised values, in an inception cohort of patients with recurrent or chronic low back pain (RCLBP).

## Methods

An inception cohort of consecutive patients presenting with a new episode of RCLBP was assembled. Eligibility criteria appear in Table 2. Patients were recruited by collaborating physiotherapists at seventeen primary care clinics and one outpatient hospital physiotherapy department, across two New Zealand provinces, for the purpose of a clinical diagnostic research study reported earlier [30]. This multi-centre cohort study research was approved by the Otago and Canterbury Regional Ethics Committees (reference # 01/05/030 & 01/10/095) of the New Zealand Ministry of Health.

A sample of volunteers with no history of low back trouble was required in order to describe normal lumbar kinematics. From this asymptomatic sample, reference ranges describing normal lumbar kinematics were established, against which the kinematic measurements of the RCLBP cohort were compared. A request for volunteers was posted on notice boards in several locations in North Dunedin, New Zealand. Eligibility criteria appear in Table 3. This asymptomatic sample project was approved by the University of Otago Human Ethics Committee.

Consenting participants completed baseline data forms, including a 10 cm visual analog pain scale and the 18 item modified Roland-Morris disability index (RM-18) [31], and were referred to radiology for flexion-extension (F-E) lateral radiographs. All female participants were screened for pregnancy by a nurse or midwife. The flexion and extension radiographs were taken with the participants standing [32]. Radiographs were taken with a source-to-film distance of 100 cm, and centred on L4. The radiographic protocol for the flexion and extension allowed the subjects to move freely. Patients were told that flexion and extension of the spine were the subject of our interest, not hip motion, however participants were unrestrained and unforced. Participants were verbally instructed to flex forward from the neck and trunk downward through the lower spine. For extension, patients were instructed to cross their arms to place each hand on the opposite shoulder, and bend backwards as far as possible. This protocol was set in order that the results should have maximal external validity for the purposes of comparison with F-E radiographs from standard clinical radiological practice. All radiographs were viewed and reported by a consultant radiologist prior to being released to the primary investigator (JHA).

For both cohorts, the radiographs were processed using a modified version of the methods of Pearcy, Bogduk and Schneider [33-36], which involves tracing the inner margin of the cortical shell of each vertebra (L2-S1), producing a matching flexion and extension pair of image tracings, and defining a trapezoidal representative image. For the asymptomatic sample this was done by the primary investigator (JHA). Intra-rater reliability, assessed using the intraclass correlation coefficient (ICC), was high for rotation (ICC_(3,1) _= 0.97, 95%CI 0.95, 0.98) and translation (ICC_(3,1) _= 0.89, 95%CI 0.80, 0.94). For the RCLBP cohort two other trained researchers (GS, RMW) contributed. Inter-rater reliability was high for both rotation (ICC_(3,1) _= 0.96, 95%CI 0.87, 0.99) and translation (ICC_(3,1) _= 0.83, 95%CI 0.47, 0.96). Radiograph tracing was performed while blinded to the clinical examination findings and radiologists' reports. Radiographs of insufficient quality to allow the analysis of two or more segments were excluded.

### Data analysis

Calculation of rotation and translation motion was performed using the Clarity *SMART *version 1.2 computer program [37], while blinded to the clinical examination findings and radiologists' reports. Concurrent validity of rotation measurement by Clarity*SMART *vl. 2 was tested against a reference standard (measurement using NIH Image [38]), and assessed using the ICC. Translation measurement was tested against manual constructions (0.3 mm pencil on tracing paper; measurements using a 0.5 mm graduated ruler). These trials demonstrated near perfect concurrence for both rotation (in degrees) (ICC_(3,4) _= 0.98, 95%CI 0.92, 0.99), and translation (in standardized units of vertebral body depth) (ICC_(3,1) _= 0.98, 95%CI 0.94, 0.99). Repeatability coefficients [39] for measurement of both rotation (2.96°) and translation (0.046) were favourably comparable with current state-of-the-art methodology [25,32,40], whose results were 2.99° and 0.034 respectively [25].

Rotation and translation values of each segment were described by mean and standard deviation (sd) values, for both the asymptomatic and RCLBP participants. The Kolmogorov-Smirnov (K-S) test was performed to evaluate conformity to a normal (Gaussian) distribution. The reference intervals for the conventional Gaussian between-subjects definition of abnormal was achieved by calculating the 2sd range for each segmental level (L2-3 to L5-S1) from the asymptomatic sample [41]. By definition, values falling below the lower bound of the 2sd range were classified as LSR. Values falling above the upper bound of the 2sd range were classified as LSI.

We then calculated the proportion of total motion (mean and sd) that is contributed by each segmental level (L2-3 to L5-S1) under normal circumstances (i.e. the asymptomatic sample). The reference intervals for the normalised within-subject definition of abnormal were defined by the 2sd range of the relative (proportional) contribution of each segmental level towards the total lumbar motion, for each of the four levels within each individual. Under this definition, we considered segments contributing significantly less than the expected proportion of motion toward total lumbar motion (i.e. below lower bound of the 2sd reference range) to have LSR, and segments contributing significantly more than the expected proportion (i.e. above the upper bound of the 2sd reference range) to exhibit LSI.

For each LSMD, prevalence was described as the proportion of segments (in the RCLBP cohort) which lay outside the reference interval [41], for both the Gaussian and normalised within-subject models. The chi squared (*χ*^2^) goodness-of-fit test was used to establish whether the number of cases falling outside the reference interval was significantly different from the number expected from a normally distributed population. Significance was set at the *p *< 0.05 level. A statistically significant result indicated an association between the LSMD and the symptom of LBP.

The data were explored for correlations between the kinematic variables and participant characteristics (age, gender, height, body mass index, disability index [31] and pain). Multiple linear regression was used to correct for conditional dependence. We also assessed the association between presence of LSMDs and disability index and pain using independent samples *t*-tests. Because a large number of *t*-tests were performed, the conventional 0.05 level of significance may lead to type I error, therefore exact *p *values are reported. All computer-assisted statistical tests were calculated using SPSS 11 for Mac OSX (Chicago, Ill., USA).

## Results

### Participants

One hundred and thirty eight (138) consenting patients with RCLBP were recruited. One hundred and eight (108) arose in primary care; the remaining 30 presented to a hospital outpatient physiotherapy department. Ten patients failed to present to radiology for F-E radiographs. Five sets of radiographs were of insufficient quality for analysis. Of the 123 included participants, 68 (55%) were males and 55 (45%) females. Further characteristics are described in [30] and Table 4.

Thirty-three individuals volunteered for recruitment into the asymptomatic sample. Three participants violated the exclusion criteria with regard to low back pain history, and were therefore ineligible. The eligible normal sample comprised of 9 (30%) males and 21 (70%) females, aged 23 to 60 years (mean 41.3, sd 12.8). Radiographic images of 3 segments (2.5%) were of insufficient quality for analysis.

### Reference ranges for sagittal rotation and translation

Mean and sd for the rotation and translation values appear in Table 5. The distribution of rotation and translation values conformed to a normal (Gaussian) distribution, with K-S z-scores ranged from 0.474 to 0.832 (all not significant, *p *= 0.493 to 0.978). It was therefore appropriate to use parametric statistics and apply the Gaussian definition of abnormality. Sections 1 & 2 of the additional files (see Additional file 1) illustrate the distribution of the data. Reference intervals for the Gaussian between-subjects definition for LSMDs appear in Table 5. Relative contribution of each segment to total lumbar motion and the reference intervals for the normalised within-subjects definition in Table 6.

### Sagittal rotation and translation in a cohort of patients with non-specific recurrent or chronic low back pain

Lumbar segmental kinematics of patients with RCLBP were more varied than those of the asymptomatic sample. Mean and sd for the rotation and translation values can be found in sections 3 through 12 of the additional files (see Additional Files 1).

### Prevalence of LSMDs under a Gaussian between-subjects model

Only 6 (1.28%) of 468 segments exhibited sagittal rotation LSI, whereas 17 (3.63%) were classified as having translational LSI. In a normally distributed sample of 468 individual segments, one would expect to see 10.67 (i.e. 2.28% of) segments in each tail, beyond the reference interval. In this cohort less than the expected number of segments were in the rotation LSI category indicating no association between sagittal rotation hypermobility and RCLBP (χ^2 ^= 2.044, critical value for 1df = 3.841). Sagittal translation LSI was significantly associated with RCLBP (χ^2 ^= 4.017, critical value for 1df = 3.841, p < 0.05).

In total, 27 (5.77%) segments were classified as sagittal rotation LSR, while 26 (5.56%) had translation LSR. Data per segment appear in Table 7. Approximately double the expected number of segments were in the LSR categories. This is statistically significant at p < 0.0005 (χ^2 ^= 25.946, critical value for 1df = 12.116). This significant difference indicates that both sagittal rotation LSR and translation LSR were associated with RCLBP.

### Prevalence of LSMDs under a normalised within-subjects model

Greater numbers of segments met a normalised within-subjects contribution-to-total-motion definition of LSMDs than a Gaussian definition (Table 8), with the exception of rotation LSR at L2-3. These indicate highly significant associations with RCLBP (*p *< 0.0005).

### Associations between the kinematic variables and participant characteristics

Total sagittal rotation and translation decreased with age (Pearson correlation *r *= -0.26, *p *= 0.007 and -0.24, 0.017 respectively), but for translation this was only significant at L2-3 (not significant at L3-4 or below). Linear regression indicated that each advancing decade predicts a 4.2° loss of total lumbar rotation range of motion (95%CI 1.2, 7.1). Neither rotation nor translation was associated with gender, height or body mass index.

Pain did not appear to be associated with sagittal segmental rotation (*r *= -0.17, *p *= 0.083), except at L2-3 (*p *= 0.001). Total translation was weakly associated with pain (*r *= -0.20, *p *= 0.044), but again was only significant at L2-3. Disability was correlated with total rotation (*r *= -0.23, *p *= 0.019), but not total translation (*r *= -0.18, *p *= 0.073). Disability was significantly associated with decrease in both variables in the upper lumbar spine (rotation to L4-5, translation only to L3-4) but not lower. As expected, pain and disability scores were highly correlated (r = 0.59, *p *< 0.001), and entering both pain and disability into a stepwise linear regression model we found that neither were significantly associated with total lumbar spinal motion.

### Association between LSMDs and RCLBP

These data suggest that LSR may be slightly more painful than other forms of RCLBP, and LSI slightly less painful than RCLBP that is not associated with a LSMD (Tables 10 &11). Only translational LSR defined under the Gaussian model reached statistical significance, with higher disability scores (mean difference 3.1, *p *= 0.010).

## Discussion

### Key findings

This study of lumbar segmental rotation and translation kinematics provides normative data based on a conventional Gaussian statistical methods, which avoids arbitrary cut-off values. We have also introduced a novel approach to diagnosing LSMDs: a normalised within-subjects contribution-to-total-motion model, which also uses sound Gaussian statistical methods, but which is intended to identify segment(s) contributing significantly more, or significantly less, to total lumbar motion, compared to other segments within the same individual. We have provided normative data and reference intervals for sagittal rotation and translation for both of these models, derived from an asymptomatic reference sample. Our methodology would appear to have high external validity for use in clinical radiology, as we use standard methodology, rather than unusual patient positioning or devices. We have used exactly the same methodology for both the reference group and the RCLBP group, and have used methodology with excellent validity and reliability [42].

LSMDs were found to be associated with presence of LBP, with LSMDs found in significantly higher prevalences in patients with RCLBP, compared to the numbers expected within a normally distributed asymptomatic population, suggesting that LSMDs are valid diagnostic entities.

The normalised within-subjects approach was more sensitive than the conventional between-subjects approach for defining LSMDs, identifying more than double the number of segments with LSMDs, particularly at the lower lumbar segments where LBP is thought to more commonly arise.

### Strengths and limitations

The major strength of our study was that it used an inception cohort of consecutive patients presenting with a new episode of recurrent or chronic low back pain, in a mostly primary care setting. Previous studies of sagittal displacement kinematics in this population have used non-probability samples, such as a convenience sample. Our asymptomatic sample had experienced no low back pain resulting in absence from work or interruption of normal activities for more than one day in the previous three years, and were well matched to the RCLBP cohort in regards to age. A potential limitation exists in regards to gender imbalance between the asymptomatic sample (70% female) and the RCLBP cohort (45% female), however our data, in concurrence with others [15], suggest that there is no significant difference in these kinematic variables between the sexes.

A limitation of the normative data we present is that it is based on a sample of only thirty asymptomatic volunteers. Replicating this research using the same methodology would provide a validation sample to verify whether these normative values are representative, and that the reference intervals are sound. We do not recommend adoption of these methods or reference values until further research has increased the pool of normative data and the new data is found to be consistent with our estimates.

If a patient is unwilling to flex or extend fully from a standing position, perhaps because of pain, fear, or apprehension, both rotation and translation values will be low, even if the patient's spine was actually capable of moving normally [25]. This type of guarding behaviour may mask LSI, leading to a false negative finding. Segmental translation and rotation, as quantitative measures of abnormal spinal kinematics, may therefore be confounded by simple unwillingness of the patient to move as much as he or she may be able, which would be a limitation of the methodology. Our data, however, indicate that pain was only weakly associated with decreased movement, if at all, and then only in the upper lumbar spine. In either event, this is the pragmatic reality of interpreting F-E radiographs in clinical practice, and our study provides data with a high level of external validity for that purpose. If a segment really is unstable, i.e. has lost its "ability to maintain its pattern of displacement of the spine under normal physiologic loads" (Panjabi 1992b), we suggest that the present methods would present a normal physiological load to test that criterion. Even if an individual is unwilling to move because of back discomfort, each segment within that individual should be expected to contribute its "fair share" to total lumbar motion. The normalised motion approach we offer here avoids that limitation.

### Lumbar segmental displacement kinematics

In concurrence with other reports in the literature, there appears to be wide variability of both sagittal rotation [7,15,21,43] and translation [7,21] in asymptomatic individuals. Total rotation motion (L2-S1) averaged 35° (sd 17°), but ranged from -0.5° to 69°. While few other reports have noted negative rotation in a normal sample, these data feature 6 segments (5%) recording negative rotation, and a further 3 (9 total, 7.7%) recording rotation <1°. The tails of translation data distribution are marked by 9 (7.7%) segments recording negative translation, including 5 (17.9%) L5-Sl segments. Few segments (4, 3%) recorded translation over 15% of vertebral body depth, or around 5–6 mm, and were all associated with rotation >14°. The 20 segments with the highest translation were all associated with rotation at or above the mean, but paradoxically, among the 20 segments with the least translation there are 3 cases with rotation >9°, and another with 8.4°. Other researchers have noted that segments rotating 0° [16] and negative translation (particularly at L5-S1) [21] are not uncommon findings in an asymptomatic population.

A point of difference from the trend seen in the literature is that, while the present data concur that the average rotation at L2-3 is around 10°, the present data see rotation decreasing with each inferior segment. Other reports generally report increasing rotation [7,15,16,21,22,25,44-47], although some find L5-S1 reduced [21,22,25,47]. This effect is likely to be due to methodological differences, wherein the protocols for many of the earlier studies involved clamping the pelvis during standing flexion-extension, applying overpressure to the trunk, or F-E radiography in the sitting position [7,21,23-25,48] which would alter forces on the spine considerably and therefore affect spinal kinematics. This study, not fixing the pelvis or imposing overpressure to the trunk, allows the natural variability inherent in spinal motion to play out normally, and make the data more generalisable to those from standard radiological practice.

### Lumbar segmental instability

Spratt *et al*, investigating the prevalence of LSI, concluded that "the initial requisite for establishing instability as a clinical syndrome was met" [13], that being a significant difference in prevalence between normals and abnormals. Many studies report high prevalence of LSI (23–69%) on F-E radiographs of subjects with chronic LBP [2,5,8,20,49]. Using arbitrary definitions of "abnormal" may, however, lead to high rates of false-positive classification (i.e. classifying a segment as having LSI, when in fact it is within a "normal" range), as seen in the high positive classification rates for asymptomatic subjects [7,16,50]. In the light of these issues, the interpretation of many of the reports of prevalence of LSI are cast into doubt.

By using statistically defensible Gaussian definitions for LSI, we reduce false positive classification in asymptomatic subjects to 2.28% per segment. In the RCLBP cohort, our prevalence rates for the conventional Gaussian between-subjects model may be lower than previous reports due to lower false-positive classifications, wide variation in the normative data, or lower severity of LBP in population from which the cohort was drawn. Dvorak *et al *(1991) used a similar conventional Gaussian approach, deriving their reference values from means calculated in a previous study of healthy volunteers [21]. They found that 9% of their sample of 101 LBP patients demonstrated rotational LSI, while only 5% demonstrated anterior sagittal translatory LSI [22]. Although these proportions are small, they both reached statistical significance. Our data indicate similar findings to Dvorak *et al *for translational LSI, but we did not find significant numbers with rotational LSI. Prevalence should not be estimated from a non-probability sample, however, so the numbers provided by Dvorak *et al*. should not be mistaken for estimates of population prevalence.

We found higher prevalence of LSI using the normalised within-subjects model, compared to a conventional Gaussian between-subjects model. Using differences models for defining LSMDs will, of course, inevitably result in different prevalence findings. Both of our models result in higher prevalences than the cut-off values proposed by White & Panjabi [12] (Table 9), which is not unexpected as their criteria were intended for a surgical population at the more severe end of the LBP spectrum, while our cohort were a conservative care, mainly primary care population. The present research cannot establish which of these models is more appropriate.

Previous research has found the cut-off values of Posner et al [51] to be useful in predicting outcome following surgical fusion [52]. Other investigators have found sagittal translatory movement to be significantly correlated to the severity of LBP in patients with spondylo- or retro-listhesis [19], and with persistent LBP in longitudinal research designs [53]. Future research might assess the predictive validity of these definitions of LSI.

### Lumbar segmental rigidity

Few research reports discuss LSR, possibly because LSI may be considered an indication for surgery (primarily spinal fusion), whereas the management of LSR may be less apparent. There is growing evidence, however, that the identification of subgroups of patients with LBP who have clinical features of LSR is useful in prescribing therapies [3,54-56].

The non-probability sample of Dvorak and colleagues [22] consisted of 101 patients with LBP of various types, including lytic spondylolisthesis, radicular syndromes, degenerative intervertebral discs, and non-specific LBP, and found LSR to be prevalent across all ages and all motion segments of all LBP groups. Similar proportions were seen in both rotation LSR and translation LSR, both far more prevalent than LSI by more than 5 to 1.

Mayer *et al*. [57] found LSR in 17% of a prospective cohort of 421 patients with chronic work-related disabling LBP referred to a tertiary rehabilitation centre, which is similar to our findings (19.6%) for rotational LSR at any level under a conventional Gaussian definition. Under the normalised within-subjects definition we found a higher prevalence (29.8% for rotational LSR, 35.5% for translational LSR), with most problems at the lower lumbar levels, a finding concurrent with Mayer's description [3]. Two studies validating a clinical prediction rule for identifying patients who respond to spinal manipulation found the prevalence of a clinical syndrome that equates to LSR to be 37% [55] and 45% [54] in their clinical cohorts. These studies indicate that the identification of this syndrome by clinical prediction rule is very useful for directing intervention [54,55].

In all definitions of LSR, pain and disability were higher on average compared to other patients with non-specific RCLBP but without LSMDs. This study was not designed to test for such differences, and due to large standard deviations and a possible floor effect (due to only moderate pain and disability in this mainly primary care cohort consulting physiotherapists) may not have sufficient power to detect a clinically important difference, should one be present. We report the data only for the information of future researchers, and did not hypothesise *a priori *that LSMDs would be expected to be any more or less painful than any other form of non-specific LBP. Only the Gaussian definition of translational LSR reached statistical significance (*p *= 0.010), and the magnitude of difference (3.1 points) could also be considered clinically significant [58]. All other values were only of modest magnitude, however, and it is possible that this could merely be a chance finding.

### Lumbar segmental mobility disorders

These data indicate that LSMDs are significantly associated with the symptom of LBP, in that they are found in significantly greater numbers in patients with RCLBP compared to an asymptomatic reference sample. These data suggest that LSMDs comprise valid sub-groups within "non-specific" LBP. Identifying valid sub-groups of low back pain has consistently been rated as the highest priority research goal, by the International Forum for Primary Care Research on Low Back Pain [59,60]. Failure to validly recognise differing sub-groups has been identified as a probable reason for poor progress in low back pain intervention research [61]. There is growing evidence that the identification of sub-groups corresponding to LSMDs, and matching the treatment accordingly, leads to better therapeutic outcomes, when interventions theoretically intended to correct the LSMD are provided [3,52,54,55,57,62-64]. These methods may be utilised within such clinical research designs. We do not advocate adopting these methods for routine clinical practice: the methodologies and reference intervals should be replicated by further research, and coupled with further evidence regarding whether diagnosing LSMDs is useful for directing interventions. Until research finds the methods convey important advantages to the patient, the economic cost and the risks of radiation exposure, while small, are unwarranted. The data provided in this research may be useful in designing future clinical research. The methods can be used for studying the validity of clinical examination procedures or clinical syndromes [30], or for identifying sub-groups of patients having greater odds of success from surgical fusion, exercise interventions intended to enhance lumbar stability, or manual therapies intended to mobilise rigid segments.

## Conclusion

In this paper we have described the sagittal displacement kinematics of an inception cohort of patients with recurrent or chronic low back pain, and an asymptomatic reference sample. We have approached the data using a conventional between-subjects Gaussian definition of abnormality (2sd from a reference mean), and have also proposed a novel definition of lumbar segmental mobility disorders using a normalised within-subjects contribution-to-total-motion model. We have provided reference intervals for normal sagittal rotation and translation for both of these approaches, and have estimated the prevalence of LSR and LSI for both definitions in a mainly primary care RCLBP population. With the exception of rotational LSI (conventional between-subjects definition), LSMDs are found in statistically significant prevalences in patients with RCLBP. Among patients with RCLBP, however, presence of any LSMD, regardless of how defined, does not appear to be strongly associated with greater levels of pain or disability compared to patients with other forms of non-specific RCLBP but without LSMDs.

## Competing interests

The author(s) declare that they have no competing interests.

## Authors' contributions

JHA conceived, designed and coordinated the study, recruited clinicians and participants, carried out data analysis and prepared the manuscript. JHA retains copyright on all contents. JMF contributed to data analysis and manuscript preparation. BMcC assisted with measurement technology & data analysis, and manuscript preparation. BS advised on statistical analysis of data. PH provided statistical support. BL provided radiological consultation and provided facilities. GS and RMW assisted with radiographic methodology and data analysis. All authors read and approved the final manuscript.

## Pre-publication history

The pre-publication history for this paper can be accessed here:



## Supplementary Material

Additional file 1 Additional file 1 Click here for file

## Figures and Tables

**Table 1:** Six methods for defining normal in biomedical science*.

method	Properties of the clinical data, sign, or test
Gaussian	The statistical distribution of the data is known to be normal in persons without the disease.
Percentile	Test result lies within a certain percentile of the possible range of results.
Diagnositic	Research has established the probability that the target disease is present, for a given range of test results.
Therapeutic	Research has shown that a specific treatment has a known probability of success for a given range of test results.
Risk factor	Research has shown that presence of a risk factor increases risk of a specified outcome (e.g. morbidity or mortality).
Culturally desirable	Carries strong socio-political expectations of normal appearance or behaviour.

Notes: *as described by Sackett et al (1997) and Smith (2002).

**Table 2:** Eligibility criteria for the RCLBP cohort

Inclusion criteria: Patients presents to clinic with a new episode of low back pain: Patient has history of recurrent or chronic lower back pain, the first episode of which occurred at least 12 weeks ago; Age over 20 years; Primary means of communication is the English language.
Exclusion criteria: Pregnancy: current intention to become pregnant; English as a second language; upper motor neurone lesion; frank psychiatric illness; traumatic fracture of any vertebra leading to permanent neurological damage or persistent disability; non-ambulatory or requiring assistive devices; any spinal surgery within six months prior to presentation.

**Table 3:** Eligibility criteria for the asymptomatic reference sample

Inclusion criteria: Age 20–59; No current low back pain or back pain-related disability; No significant back pain (resulting in absence from work or interruption of normal activities for more than one day), in the last three years. Primary means of communication is the English language.
Exclusion criteria: Pregnancy; current intention to become pregnant; Any history of low back pain, which resulted in absence from work or interruption of normal activities for more than one day, in the last three years; upper motor neuron lesion; frank psychiatric illness; traumatic fracture of any vertebra leading to permanent neurological damage or persistent disability; non-ambulatory or using devices to assist gait; known spondylolysis or spondylolisthesis; any spinal surgery.

**Table 4:** Description of the RCLBP cohort

	Mean	sd	Range	N
Age	40.0	11.2	20–75	106
Body mass index	26.7	4.75	19.8–43.0	85
Years since first LBP episode	8.3	8.0	<1–33	104
Disability score (out of 18)	7.13	4.543	0 – 17	119
Pain level (out of 100)	42.7	25.7	0 – 100	117
Proportion with constant LBP	.23	.420	-	106

Notes: RCLBP = recurrent or chronic low back pain. sd = Standard deviation; N = number with complete data. Disability score was assessed on the modified Roland-Morris RM18 (Stratford *et al*. (1997); Pain level was patient-rated on a horizontal 10 cm visual analog scale.

**Table 5:** Kinematic data for each segment, and reference intervals for diagnosis of LSMDs under a Gaussian between-subjects model from asymptomatic sample (n = 30).

	Rotation		Translation	
	Mean (sd)	Reference interval	Mean (sd)	Reference interval

L2 – 3	10.7(4.56)	1.63, 19.87	.079 (.04)	-.002, .159
L3 – 4	10.0 (4.83)	0.34, 19.68	.066 (.04)	-.022, .148
L4 – 5	8.2 (5.31)	-2.44, 18.79	.058 (.03)	-.011, .123
L5 – S1	6.8 (6.25)	-5.71, 19.29	.032 (.04)	-.046, .109

Notes: Rotation data are measured in degrees. Translation data are measured in units of vertebral body depth. The radiographic image of one L2-3 segment and 2 L5-S1 segments were of insufficient quality for analysis. Reference interval is defined by 2 standard deviations either side of the mean.

**Table 6:** Proportional contribution of each segment normalised to total lumbar motion

	Rotation		Translation	
	Mean (sd)	Reference interval	Mean (sd)	Reference interval

L2 – 3	.302 (.158)	-.015, .619	.331 (.147)	.037, .625
L3 – 4	.292 (.082)	.120, .463	.262 (139)	-.015, .539
L4 – 5	.221 (.106)	.009, .433	.249 (.118)	.012, .485
L5 – S1	.186 (.161)	-.136, .507	.158 (.175)	-.191, .508

Notes : Rotation data are measured in degrees. Translation data are measured in units of vertebral body depth. Reference interval is defined by 2 standard deviations either side of the mean.

**Table 7:** Prevalence (%) of LSMDs in the RCLBP cohort under a Gaussian between-subjects model

	Rotation		Translation	
	LSR	LSI	LSR	LSI
L2 – 3	15.8	0	5.3	1.8
L3 – 4	5.0	0	2.5	1.7
L4 – 5	0.9	3.4	7.8	6.9
L5 – S1	1.0	1.7	6.0	4.3
Any level	19.6	4.7	17.8	12.1

Notes: LSR = lumbar segmental rigidity; LSI = lumbar segmental instability. Calculation of LSMD at 'any level' was only performed in cases in which all four motion segments were analysed. No patient had both LSR and LSI of either rotation or translation.

**Table 8:** Prevalence (%) of LSMDs in the RCLBP cohort under a normalised within-subjects model

	Rotation		Translation	
	LSR	LSI	LSR	LSI
L2 – 3	2.8	8.4	7.5	14.0
L3 – 4	13.1	4.7	7.5	4.7
L4 – 5	10.3	8.4	16.8	13.1
L5 – S1	6.5	7.5	13.1	8.4
Any level	28.9	23.3	35.5	31.8

Notes : LSR = lumbar segment rigidity; LSI = lumbar segmental instability; Calculation of LSMD at 'any level' was only performed in cases in which all four motion segments were analysed. 20 patients (18.7%) had both rotational LSR and rotational LSI, 26 patients (24.3%) had both translational LSR and translational LS.

**Table 9:** Prevalence (%) of segments exceeding criterial suggested by White & Panjabi (1990)

	Rotation	Translation
	LSI	LSI
L2 – 3	7	1.8
L3 – 4	9.2	1.7
L4 – 5	1.7	2.6
L5 – S1	0	2.6
Any level	15.0	7.5

Notes: LSI = lumbar segmental instability.

**Table 10:** Association between presence of LSMDs and pain & disability scores – Gaussian between-subjects model

	VAS pain		RM – 18	
	
(n)	Mean (sd)	Mean diff. Sig.	Mean (sd)	Mean diff. Sig.
No LSMD (75)	4.1 (27.0)	0.6	6.6 (4.6)	1.5
Rotational LSR (22)	4.7 (22.9)	.325	8.2 (4.6)	.156
No LSMD (72)	4.2 (26.3)	0.4	6.5 (4.5)	3.1
Translational LSR (17)	4.6 (30.7)	.547	9.7 (4.3)	.010*
No LSMD (75)	4.1 (27.0)	-1.2	6.6 (4.6)	-0.6
Rotational LSI (5)	2.9 (28.1)	.338	6.0 (4.5)	.760
No LSMD (59)	4.2 (26.3)	-0.7	6.5 (4.6)	-1.0
Translational LSI (8)	3.5 (18.6)	.363	5.5 (3.8)	.465

Notes: LSMD = lumbar segmental mobility disorders; (n) = number in group; (sd) = standard deviation; Sig. = 2-tailed significance of independent samples *t*-test; VAS pain = visual analog scale pain score (cm); RM-18 = 18 item modified Roland-Morris disability index score (Stratford *et al*. 1997); LSR = lumbar segmental rigidity; LSI = lumbar segmental instability; *statistically significant at *p *< .01.

**Table 11:** Association between presence of LSMDs and pain & disability scores – normalised within-subjects model.

	VAS pain		RM – 18	
	
(n)	Mean (sd)	Mean diff. Sig.	Mean (sd)	Mean diff. Sig.
No LSMD (68)	4.0 (25.1)	0.4	6.4 (4.4)	2.7
Rotational LSR (11)	4.4 (29.3)	.609	9.0 (4.4)	.068
No LSMD (59)	4.1 (26.0)	0.1	6.5 (4.3)	1.9
Translational LSR (12)	4.2 (26.1)	.962	8.3 (4.6)	.179
No LSMD (68)	4.0 (25.1)	-1.2	6.4 (4.4)	-1.2
Rotational LSI (11)	2.8 (24.9)	.305	5.2 (5.2)	.578
No LSMD (59)	4.1 (26.0)	0.6	6.5 (4.2)	-0.9
Translational LSI (8)	4.8 (26.8)	.516	5.6 (5.2)	.607

Notes: LSMD = lumbar segmental mobility disorders; (n) = number in group; (sd) = standard deviation; Sig. = 2-tailed significance of independent samples *t*-test; VAS pain = visual analog scale pain score; RM-18 = 18 item modified Roland-Morris disability index score (Stratford *et al*. 1997); LSR = lumbar segmental rigidity; LSI = lumbar segmental instability
