# Supplementary material for: Lumbar segmental mobility disorders: comparison of two methods of defining abnormal displacement kinematics in a cohort of patients with non-specific mechanical low back pain
Source: BMC Musculoskelet Disord. 2006 May 19;7:45. doi: 10.1186/1471-2474-7-45 (PMC1550717; doi:10.1186/1471-2474-7-45)

Additional files

Section 1 - Distribution of sagittal rotation data for all segments (L2-S1) of the asymptomatic reference sample, with a Normal probability curve overlaid.

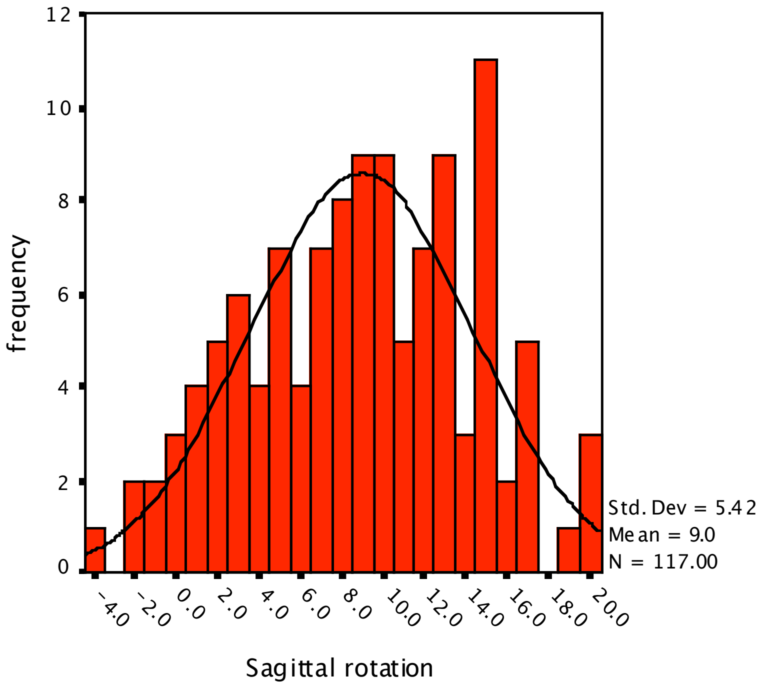

Section 2 - Distribution of sagittal translation data for all segments (L2-S1) of the asymptomatic reference sample, with a Normal probability curve overlaid.

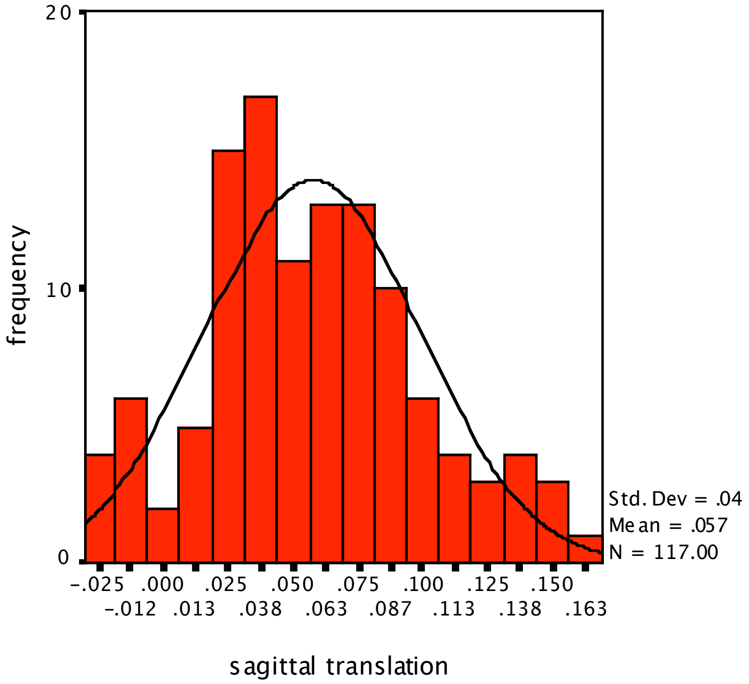

Section 3 - Distribution of sagittal rotation mobility data for all segments (L2-S1) from the RCLBP cohort, with a Normal probability curve overlaid.

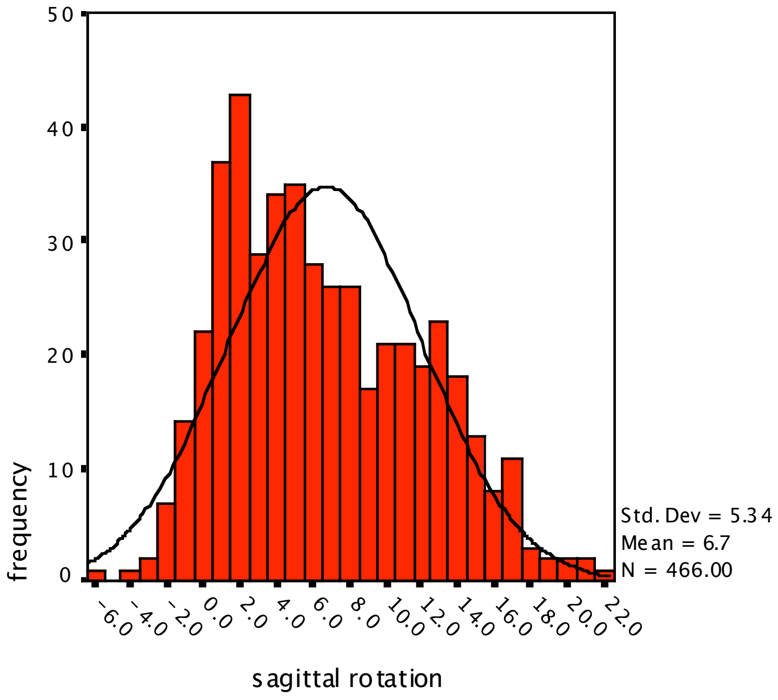

Section 4 Distribution of sagittal translation mobility data for all segments (L2-S1) from the RCLBP cohort, with a Normal probability curve overlaid.

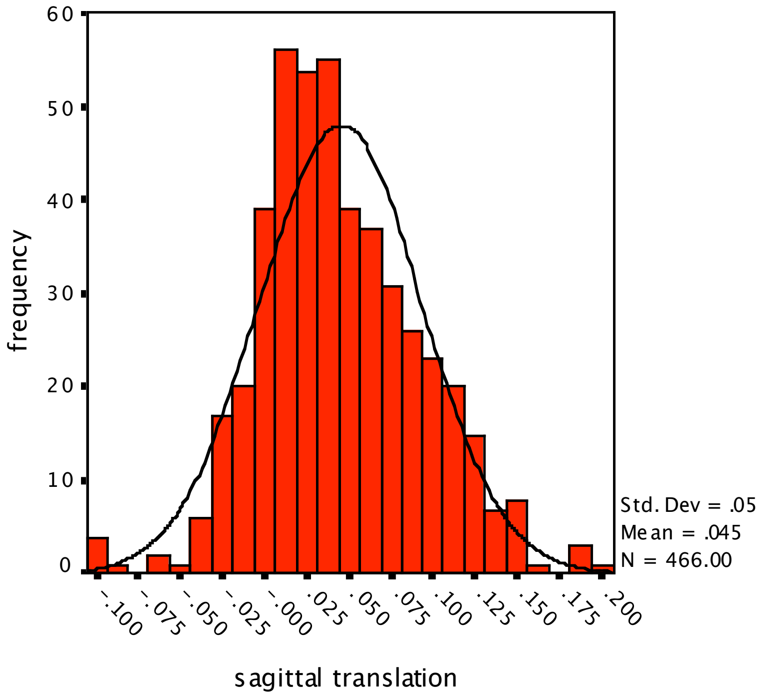

Section 5 – Rotation L2-3: RCLBP cohort

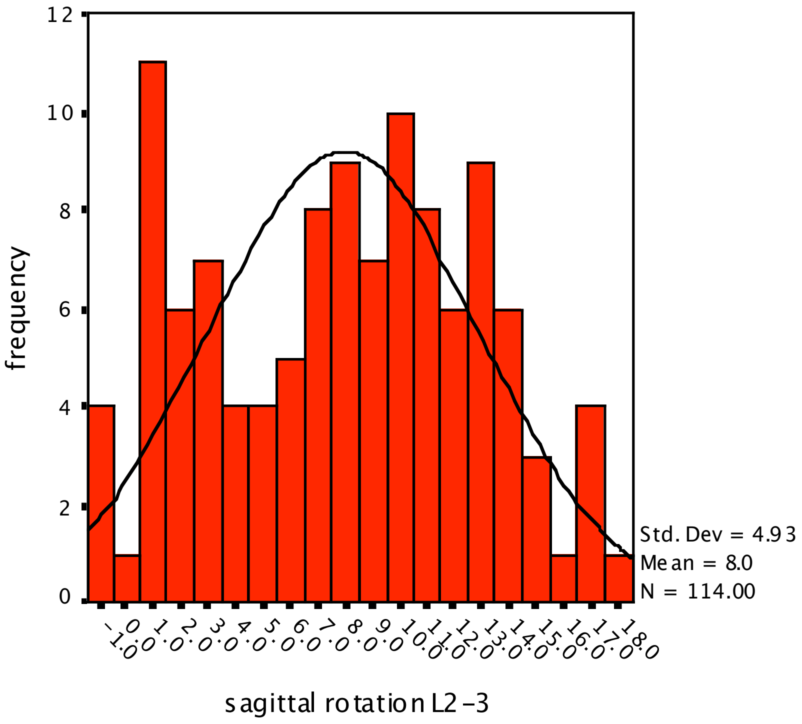

Section 6 - Translation L2-3: RCLBP cohort

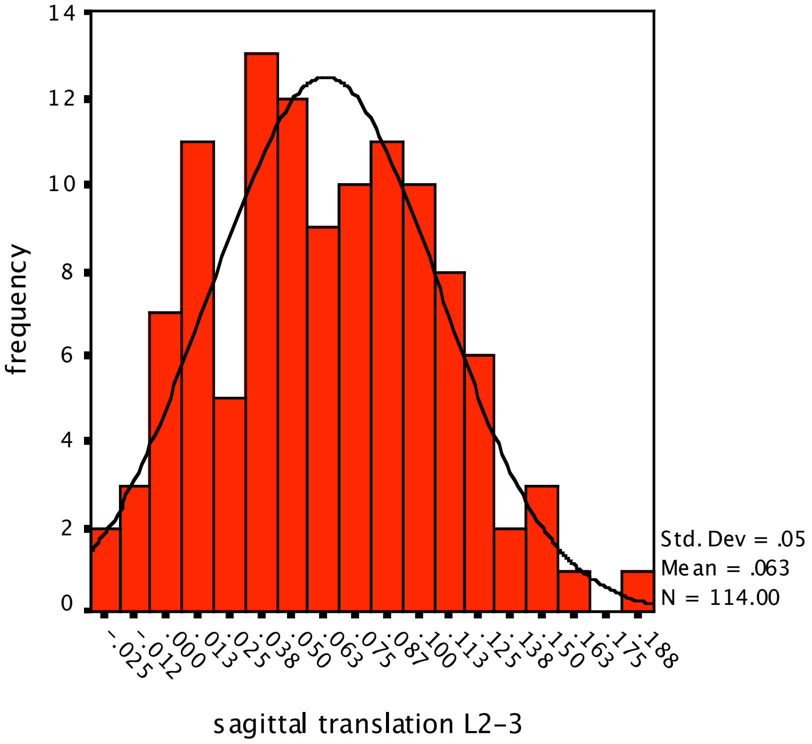

Section 7 - Rotation L3-4: RCLBP cohort

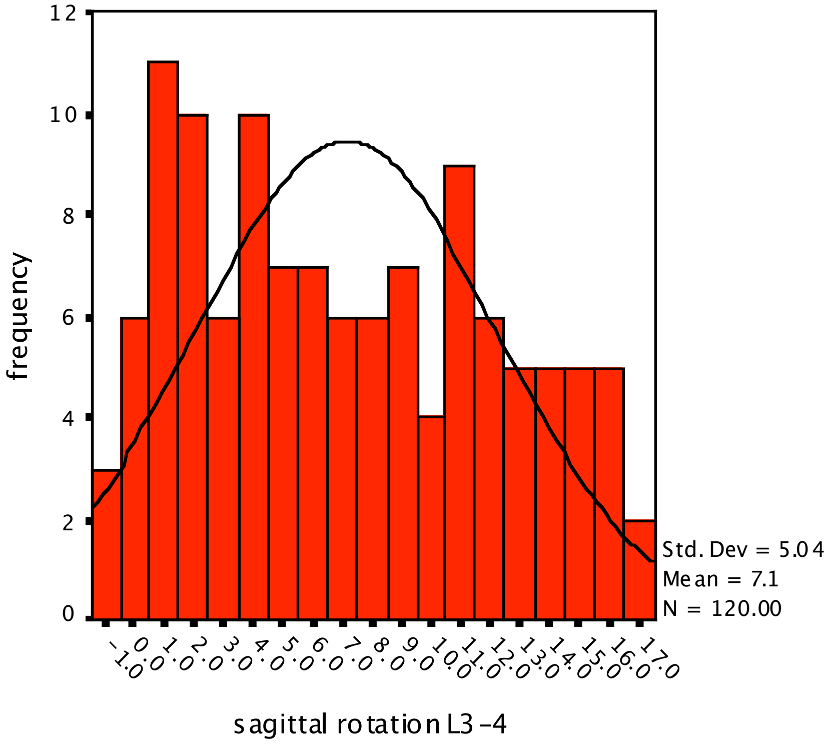

Section 8 - Translation L3-4: RCLBP cohort

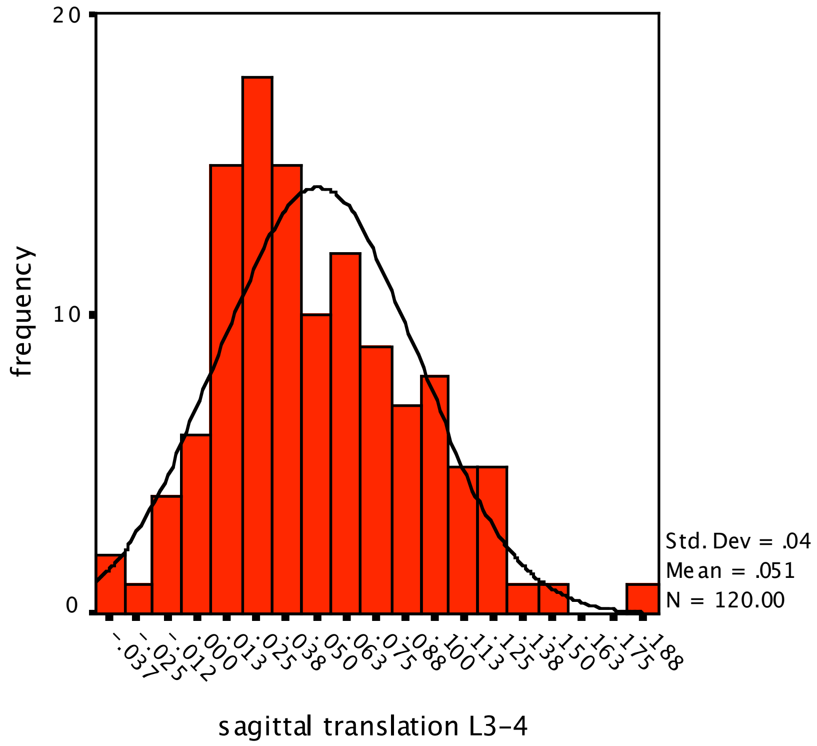

Section 9 - Rotation L4-5: RCLBP cohort

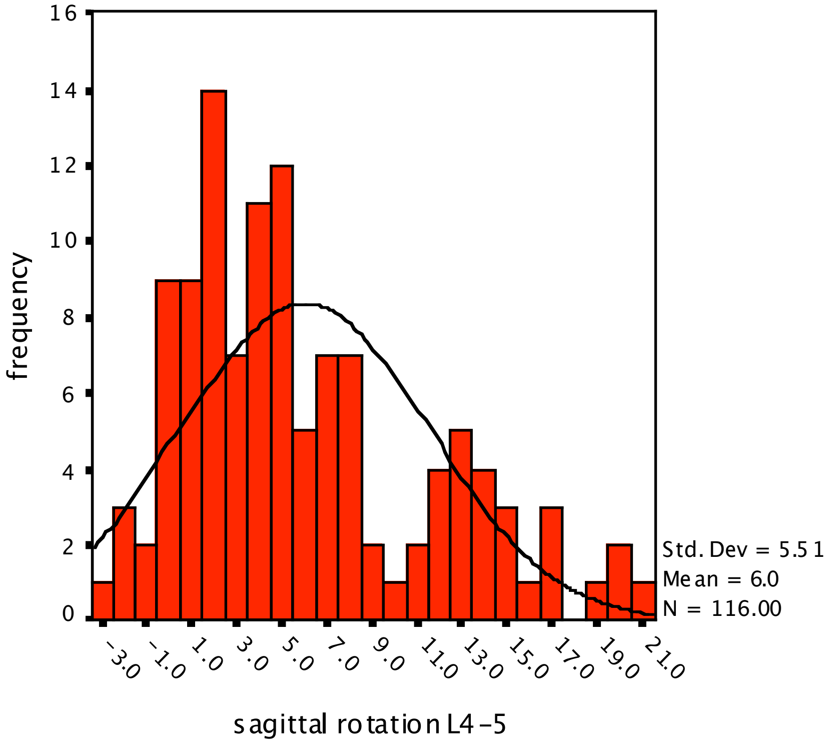

Section 10 - Translation L4-5: RCLBP cohort

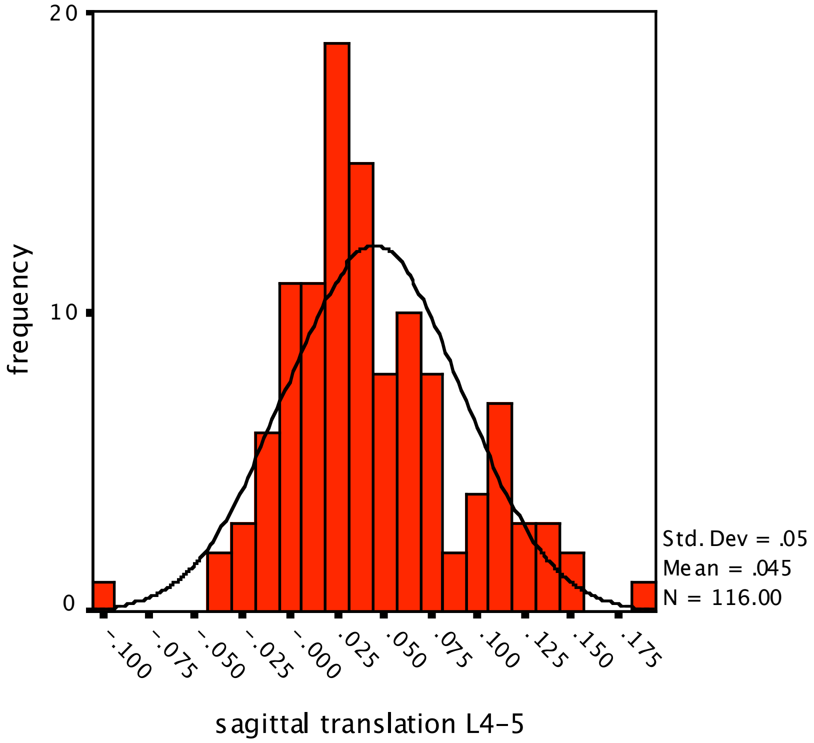

Section 11 - Rotation L5-S1: RCLBP cohort

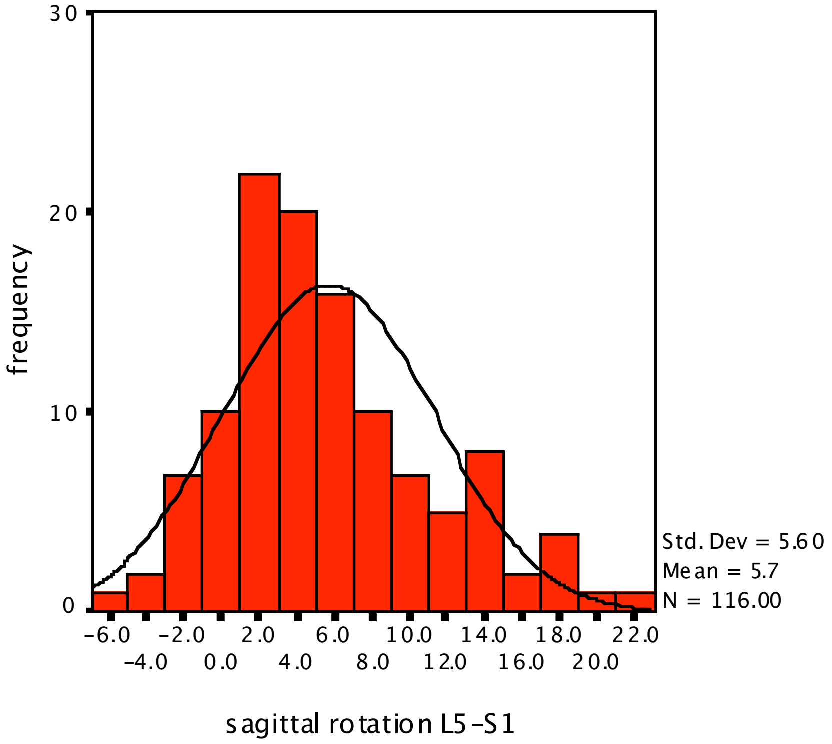

Section 12 - Translation L5-S1: RCLBP cohort

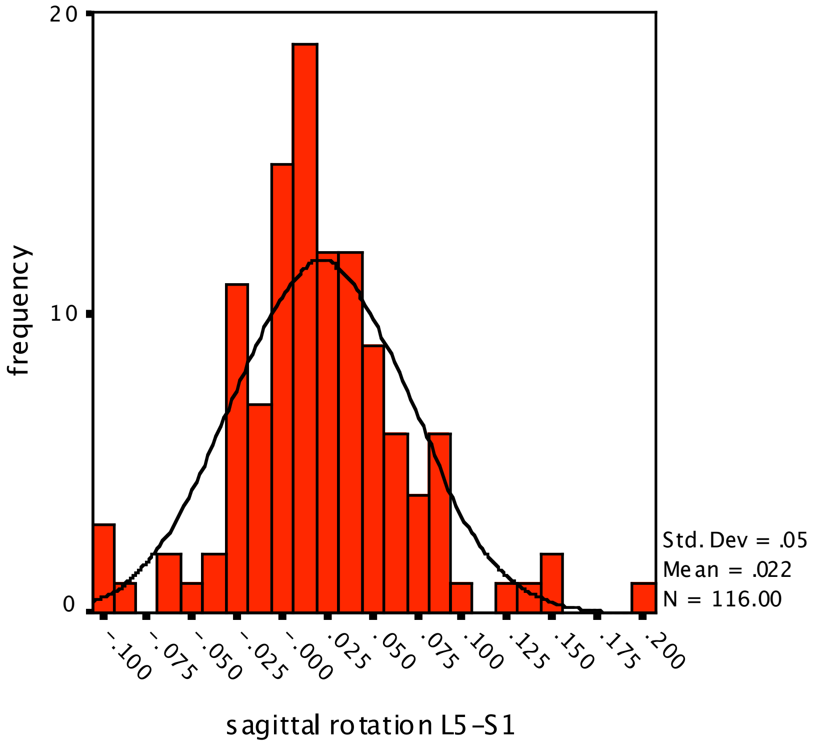

Supplement: Additional file 1 [file 1471-2474-7-45-S1.pdf]
